# A dynamic bid price approach for the seat inventory control problem in railway networks with consideration of passenger transfer

**DOI:** 10.1371/journal.pone.0201718

**Published:** 2018-08-15

**Authors:** Wuyang Yuan, Lei Nie, Xin Wu, Huiling Fu

**Affiliations:** 1 School of Traffic and Transportation, Beijing Jiaotong University, Beijing, China, 100044; 2 State Key Laboratory of Rail Traffic Control and Safety, Beijing Jiaotong University, Beijing, China, 100044; Lanzhou University of Technology, CHINA

## Abstract

Railway seat inventory control aims to maximize ticket sale profits by determining a selling policy on the reservation horizon. This paper introduces a dynamic bid price approach in railway seat inventory control problem. Multi-dimensional demand is taken into consideration in modeling the problem, in which passenger transfer is our main focus. A new approximate approach is designed to this problem. Numerical examples are presented to evaluate the efficiency of this approach. Simulation experiments are conducted to verify the impact of transfer under different scenarios.

## 1 Introduction

Ticket reservation is an important step in railway operation management. It determines the final income of a railway enterprise. Seat inventory control, as one of the core issues in ticket reservation, aims to identify a “good” selling policy to maximize total revenue with fixed supply.

Seat inventory control belongs to the typical research topic of revenue management (RM), which dates back to the deregulation of the domestic airline industry in the U.S. in the 1970s, and its application has reached the airline, hotel and railway industries. The research on railway seat inventory control has received the attention of railway operators in multiple countries, including Amtrak [1], SNCF [2] and CRC (China Railway Corporation) [3, 4].

**Fig 1:**
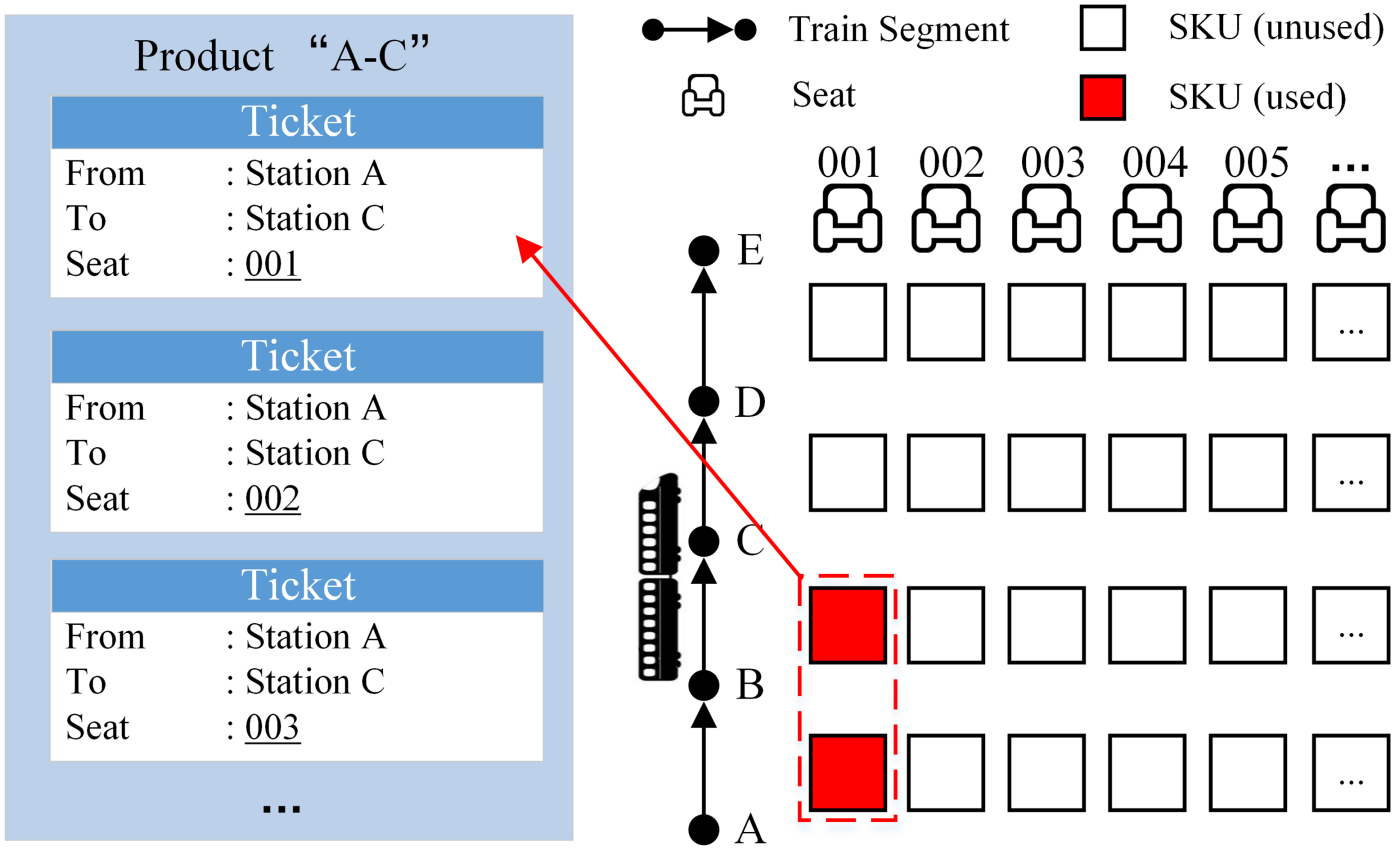
Basic Elements in the seat inventory control problem.

Some definitions in railway seat inventory control are listed below. And an example is given in Fig 1, in which a train starts from station A and stops at a sequence of intermediate stations before the terminal station E.

- **Train segment**
  A train segment is the trip of a specific train within two adjacent intermediate stations.
- **Seat-keeping unit (SKU)**
  A seat-keeping unit is the lowest level unit of inventory, which refers to a specific seat over a specific train segment. In Fig 1, each square represents a SKU.
- **Ticket**
  A train ticket, which contains information of the train number, the seat number, the origin station, the destination station, etc., can be interpreted as the entitlement to one or more SKUs. The ticket in Fig 1 from station A to station C of Seat No.001 occupies the two marked SKUs.
- **Product**
  A product is a class of tickets with the same train ID, origin station, destination station and fare.
- **Offered Product**
  At some time point in the ticket booking horizon, the products which are offered to customers is called the offered product. (i.e., just a request for one of these products will be accepted).
- **Seat inventory control policy**
  A seat inventory control policy is a prepared rule/strategy, following which the offered products are decided. The inputs of a policy will include the information of remaining SKUs, short-term demand forecasting and some other factors.

During the ticket booking process, the amount of trains and seats in each train is limited. To improve total revenue, the railway operators seek for a well-designed seat inventory control policy based on the forecasted travel demand. In general, there are a variety of control policies one can determine the availability of each product. But from the perspective of practice, a common and intuitive type of policy is the SKUs are assembled into tickets before the booking horizon. This type of policy is called partitioned booking limit control, is intuitive and easy to perform. A large percentage of research has focused on this type of control [3–6].

However, partitioned booking limit control is inefficient when demand is stochastic [7]. To overcome this deficiency, adaptive control approaches, including nesting booking limit control [8] and dynamic bid price control, are implemented to have more flexibility when allocating the SKUs.

The basic idea of bid price control is to offer a product when its fare is greater than its opportunity cost. The opportunity cost of a product is calculated as the sum of a pre-defined threshold value (called the bid price) of each train segment [9]. Bid price control is first proposed in the background of airline industry, but it is also potential for application in railway enterprises. This paper focuses on an extended version of bid price control, called the dynamic bid price control, in which bid prices are updated over time with the estimation of travel demand based on the information of demand forecasting.

As increasing amounts of multi-dimension datasets are collected by railway enterprises, refined features of travel demand are considered in seat inventory control. There are three features mainly concerned.

- The randomness in the booking amount
  The randomness in the booking amount is taken into consideration since the early studies of revenue management [2, 10]. It is commonly described by a distribution for each ODF (origin-destination-Fare) [5, 6].
- The randomness of arrival time of each passenger along the reservation horizon
  The arrival time indicates the time a passenger starts to reserve tickets. The randomness of arrival time can be considered together with the booking amount. The ticket booking process is then modeled as a Poisson process [11] and its parameters can be calibrated from panel data [12].
- The heterogeneous passenger choice
  In the studies of recent years, heterogeneous passenger choice is recognized as a important factor which leads to correlation between the demand for each ticket [13]. To consider the heterogeneity, market segmentation is always introduced. The entire demand is divided into several market segments in which the customers have the same behavior patterns [14].
  It is worth noting that, transfer itineraries are more often chosen by railway passengers. In railway networks, a large percentage of passengers cannot complete their journey on a single train (There are no direct trains or the tickets of direct trains are sold out). The corresponding high percentage of transfer demand has a non-negligible impact. It is widely considered in operations research in the railway industry, including timetabling [15, 16] and line planning [17]. However, in the survey of railway RM, more focus is given to passenger choice among trains; transfers are usually not considered.

In this paper, we attempt to apply dynamic bid price control into railway seat inventory control with consideration of multi-dimension demand. An new approach is designed to solve the bid prices under the randomness of arrival time and booking amount. Passenger transfer will be specially modeled and numerical experiments will be processed to evaluate its effect.

The remainder of this paper is organized as follows. Section 2 reviews the methodologies in seat inventory control problems. Section 3 states our problem and formulates a DP model. Section 4 applies approximate approaches, including LP-based ADP and DD, to solve the problem. Section 5 presents numerical experiments and simulations. We summarize this study and propose plans for future research in Section 6.

## 2 Literature review

Bid price control was firstly proposed in the study of airline revenue management. The bid prices were calculated as shadow prices of a deterministic linear programming (DLP) model [9]. The resulting bid prices had to be updated repeatedly by solving the DLP model multiple times in the booking horizon. Lee and Hersh [11] formulated a dynamic programming (DP) model to compute dynamic bid prices, which were recorded in a prepared look-up table. Talluri and Ryzin [18] demonstrated the sub-optimality of dynamic bid price control and provided an asymptotic analysis.

### 2.1 Integrated modeling with passenger behavior

The integration of modeling seat inventory control and passenger choice has been reported in many studies. Gallego et al. [19] extended the DLP model with customer choice behavior and proposed an approximate choice-based linear programming (CDLP) model. Talluri and Ryzin [14] embedded a general discrete choice model into the DP model and found the special “efficient set” form of the optimal solution.

**Fig 2:**
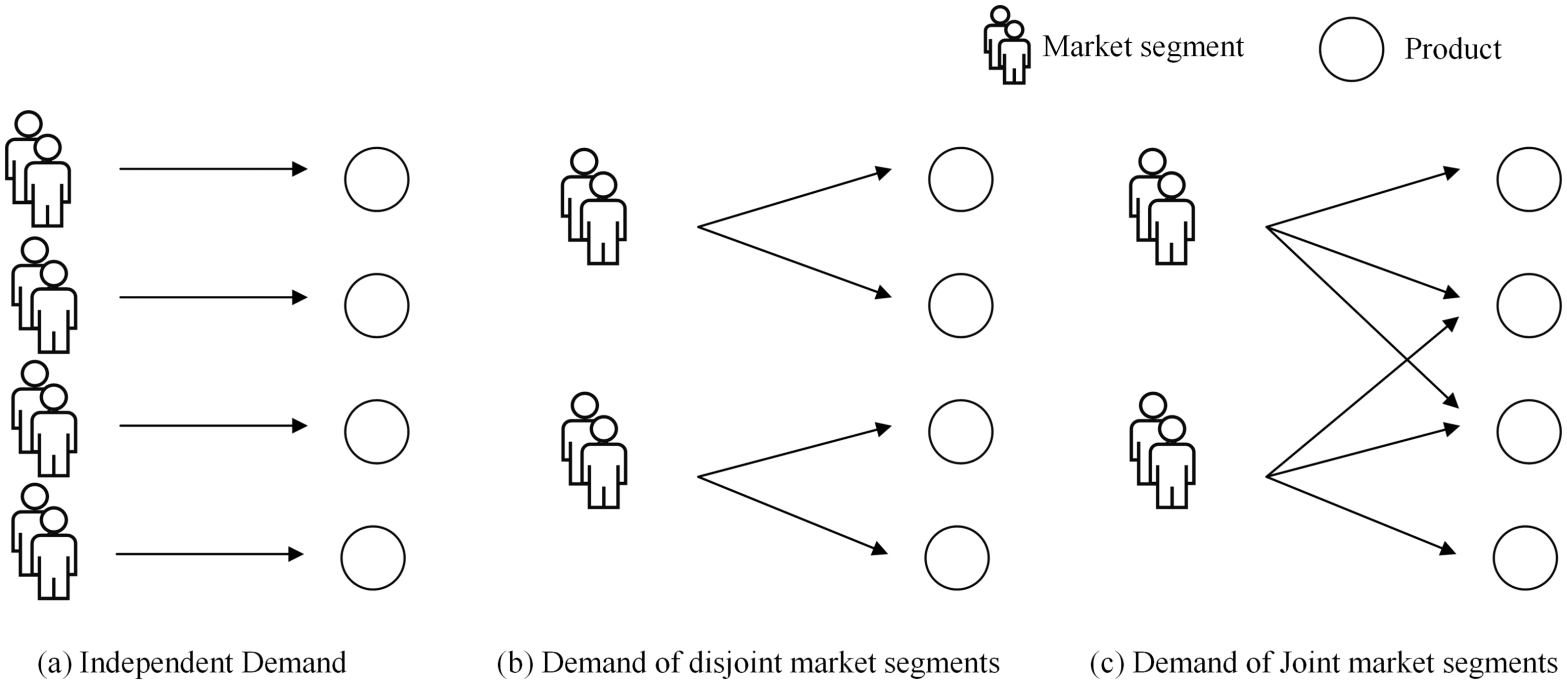
Three patterns of demand in airline seat inventory control.

In the early studies, the assumption of independent demand (Fig 2a) is alway made. When market segmentation and passenger choice behavior are introduced, customers from each market segment are modeled to choose from a set of alternative tickets (the consideration set). Liu and Van Ryzin [20] applied a multinomial logit (MNL) model with disjoint market segments (Fig 2b) and solved the problem using the column generation method. For the joint segment condition (Fig 2c), Bront and Vulcano [21] showed that the subproblem is NP-hard and developed a greedy heuristic method to overcome its complexity. Hosseinalifam et al. [22] proposed another heuristic method in combination with the Dinkelbach algorithm.

When traveling by airline, a passenger usually chooses a joint ticket to get transfer. In the modeling of airline seat inventory control, a transfer is viewed as an independent product and the problem can be transformed into the typical single-ticket-travel framework [23]. But in a railway network, transfer itineraries are more often decided by passengers themselves (buying two or more tickets simultaneously). Thus, we cannot model our problem the same as in airline field.

### 2.2 Approximate solution methods

Compared with point-to-point airline transportation, more origin-destinations (ODs) are covered by a railway network. The majority of itineraries in a railway network is composed of multiple adjacent train segments. These interrelations lead to a deep association of bid prices, which makes it difficult to estimate appropriate bid prices [2]. Moreover, railway lines cover more cities than airlines, especially in Europe and China. The large network results in more types of tickets and a larger scale of possible itineraries, which are components of the input when computing bid prices and inevitably increase the solving time.

The DP model results in substantial difficulty in solving the problem due to the large scale of the state space, which is called “the curse of dimensionality”. Approximate dynamic programming (ADP) can be applied to overcome the computational complexity. Adelman [24] introduced the affine approximate function and transformed the DP model into a tractable linear programming (LP) form with an affine function approximation, which is called the LP-based ADP model. Huang and Liang [25] proposed an exponential approximate function based on the structural properties of seat inventory control problems. Meissner and Strauss [26] extended the linear approximate function with inventory-sensitive bid prices. Vossen and Zhang [27] proposed a unified framework based on reduction of the LP-based ADP model. The complexity of the state dimensions was successfully addressed in their solution framework. Topaloglu [28] further proved the reduced form using Lagrangian relaxation.

One obstacle to computing bid prices using the LP-based ADP model is the large number of subproblems produced by the column generation method. Vossen and Zhang [29] proposed a dynamic disaggregation (DD) approach to decrease the number of subproblems. The basic idea of DD is to aggregate the subproblems in the time horizon. They reported that the solving process can be accelerated without loss of optimality if a threshold time is identified. We follow this idea and apply the DD approach to a non-homogeneous arrival situation.

### 2.3 Our contribution

Compared to the major characteristics of several papers on seat inventory control in Table 1, this study makes two contributions in dynamic bid price control for railway seat inventory control problem.

1. Compared to typical studies in railway field [4, 5], the randomness of arrival time and booking amount are considered. It leads to a non-homogeneous Poisson arrival process in modeling th e problem. To handle this, an extended version of dynamic disaggregated (DD) approach is designed. The performance of our approach is tested by a numerical example, compared with two benchmarks.
2. Passenger transfer is our main concern in modeling passenger choice behavior. Compared to the typical discrete choice models in the background of airline, passengers’ itinerary is extend to contain two or more tickets. We use simulations to verify the necessity of considering passenger transfer under dynamic bid prices control.

**Table 1:** Characteristics of various recent studies of seat inventory control.

Paper	Background	Type of Control	Solution Method	Passenger Choice Model	Transfer
Williamson [9]	Airline	Static bid price	(Not Mentioned)	Independent demand	√
Liu and Van Ryzin [20]	Airline	Static bid price	CG	MNL with disjoint segment	√
Bront and Vulcano [21]	Airline	Static bid price	CG & Heuristic	MNL with joint segment	√
Zhang and Adelman [30]	Airline	Dynamic bid price	LP-based ADP & CG	MNL	√
Topaloglu [28]	Airline	Dynamic bid price	Lagrangian Relaxation	Independent demand	√
Meissner and Strauss [26]	Airline	Dynamic bid price	LP-based ADP & CG	MNL with disjoint segment	√
Vossen and Zhang [27]	Airline	Dynamic bid price	LP-based ADP & DD & CG	Independent demand & MNL	√
Hosseinalifam et al. [22]	Airline	Dynamic bid price	CG & Dinkelbach Heuristic	MNL with joint segment	√
Ciancimino et al. [5]	Railway	Partitioned Booking limit	(Not Mentioned)	Independent demand	×
Wang et al. [4]	Railway	Partitioned Booking limit	(Not Mentioned)	MNL	×
This Paper	Railway	Dynamic bid price	LP-based ADP & DD & CG & Heuristic	MNL	√

CG = Column Generation or Constraint Generation

## 3 Problem statement

In this section the railway seat inventory control process is modeled with these three assumptions:

- Passengers have no preference on the seat allocation (they do not care if their seat is by the window or aisle).
- The seat is assigned once a reservation request is accept automatically by the computer reservation system (CRS).
- No Joint ticket is sold.

**Fig 3:**
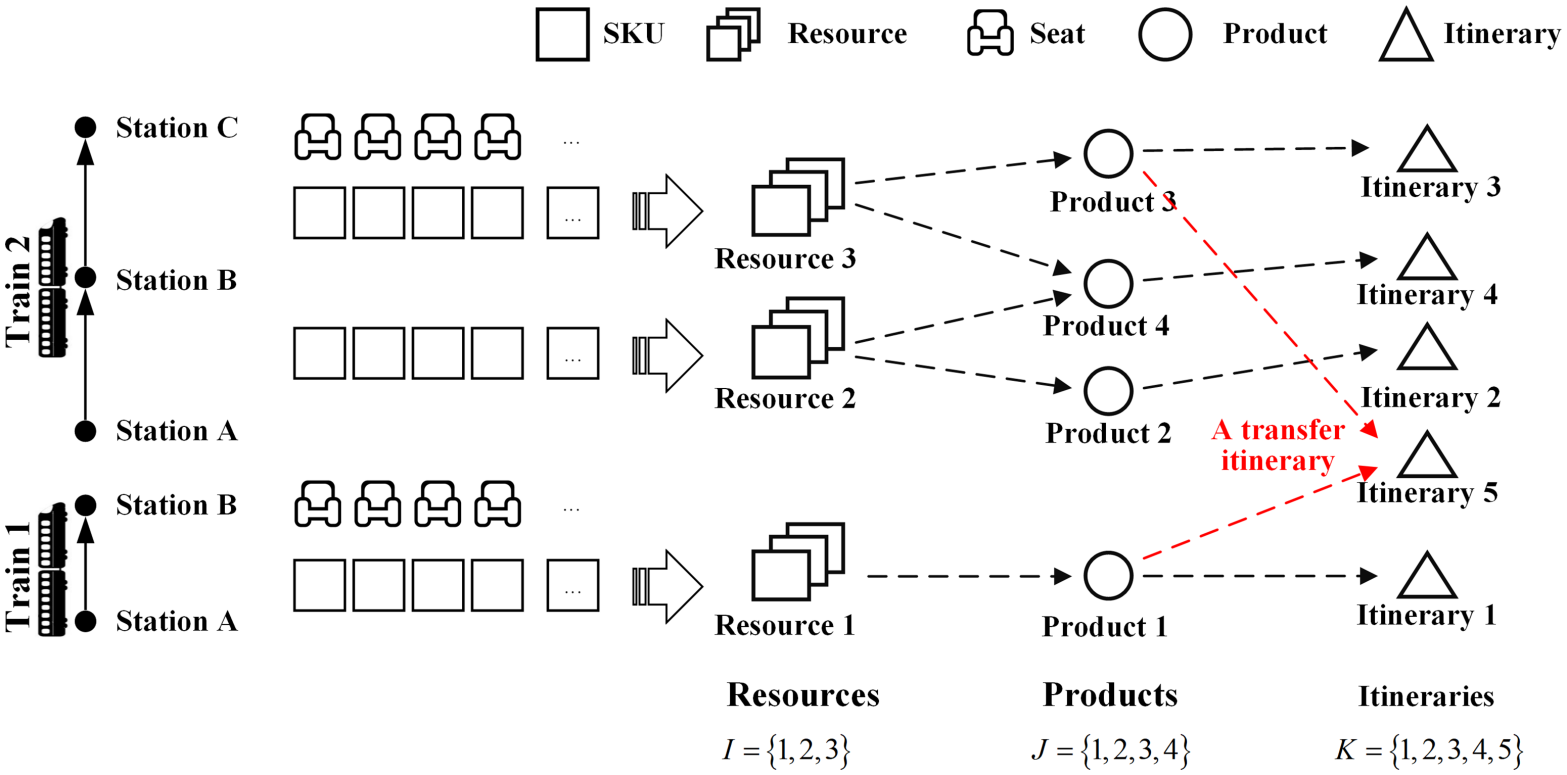
Elements in the example network.

Some extensions and simplifications of the basic elements given in Section 1 are also made in this section. To make it clear, a simple railway network of two trains is given in Fig 3.

### 3.1 Resource

With the first two assumptions, the SKUs of a same train segment are homogeneous. So that we can view them as a whole. Let us define a resource is a set of SKUs of the same train segment. This simplification is widely applied [26, 30, 31].

Let *I* denote the set of resources in the network, which is indexed by *i*. Table 2 lists three resources in the example network. Note that Resources 1 and Resource 2 are different, although they are both from A to B. Because they are provided by different trains. The amount of unused SKUs of resource *i* (or we say the remaining units of resource *i* Without causing confusion) at time *t* is denoted by *x*_*t*,*i*_. Let **x**_*t*_ denote the vector of the remaining units of all resources at time *t*. The initial vector is denoted by **c**.

**Table 2:** List of resources in the example network.

Index	Train	Origin	Destination
1	Train 1	A	B
2	Train 2	A	B
3	Train 2	B	C

Let *J* denote the set of products indexed by *j*. The fare of product *j* is denoted by *f*_*j*_. In general, a product takes at most one unit of any resource; therefore, a product can be defined as a set of resources. Table 3 shows four products in the network.

**Table 3:** List of products in the example network.

Index	Origin	Destination	Resource	Fare
1	A	B	1	40
2	A	B	2	50
3	B	C	3	60
4	A	C	2,3	80

### 3.2 Itinerary

An itinerary may contains one single product or a combination of products. Let *K* denote the entire set of itineraries, indexed by *k*. We assume that itineraries are non-overlapping, i.e. there no two identical products in a itinerary. Let **A** denote the resource-itinerary incidence matrix. If resource *i* is used by any product of itinerary *k*, then *a*_*i*,*k*_ = 1; otherwise, *a*_*i*,*k*_ = 0. The *k*th column of A is represented by *A*^*k*^, which is the set of resources occupied by itinerary *k*. The revenue of itinerary *k* is $\sum_{j\in k}f_{j}$.

Table 4 shows the itineraries in the example network. Note that Itinerary 5 is a transfer itinerary; passengers who choose Itinerary 5 will transfer from Train 1 to Train 2 at station B.

**Table 4:** List of itineraries in the example network.

Index	Origin	Destination	Products	transfer	Fare
1	A	B	1	No	40
2	A	B	2	No	50
3	B	C	3	No	60
4	A	C	4	No	80
5	A	C	1,3	Yes	100

### 3.3 The mechanism of bid price control

With the previous simplification, bid price control turns to determine the offer state of each product by assigning a bid price to each resource. Let *π*_*t*,*i*_ denote the time-dependent bid price of resource *i* at time *t*. Product *j* is open when $f_{j}\geq \sum_{i\in j}\pi_{t,i}$ and *x*_*t*,*i*_ ≥ 0 ∀*i* ∈ *j*.

To illustrate the mechanism of bid price control, assume the bid prices for the resources in the example are (30, 10, 65). Assume there is a sufficient amount of remaining resources; thus, the offer state of each product depends on only the bid price. Table 5 compares the fare (column “Fare”) and sum of the bid price of the contained resources (column “Bid price”). Thus, the state of each product is determined.

**Table 5:** State of the products in the example network.

Product	Fare	Bid price	State
1	40	30	Offered
2	50	10	Offered
3	60	65	Not Offered
4	80	10+65 = 75	Offered

Therefore, the availabilities of itineraries are determined simultaneously. Itinerary 1,2 and 4 are available, whereas itineraries 3 is not.

### 3.4 Representation of demand

**Fig 4:**
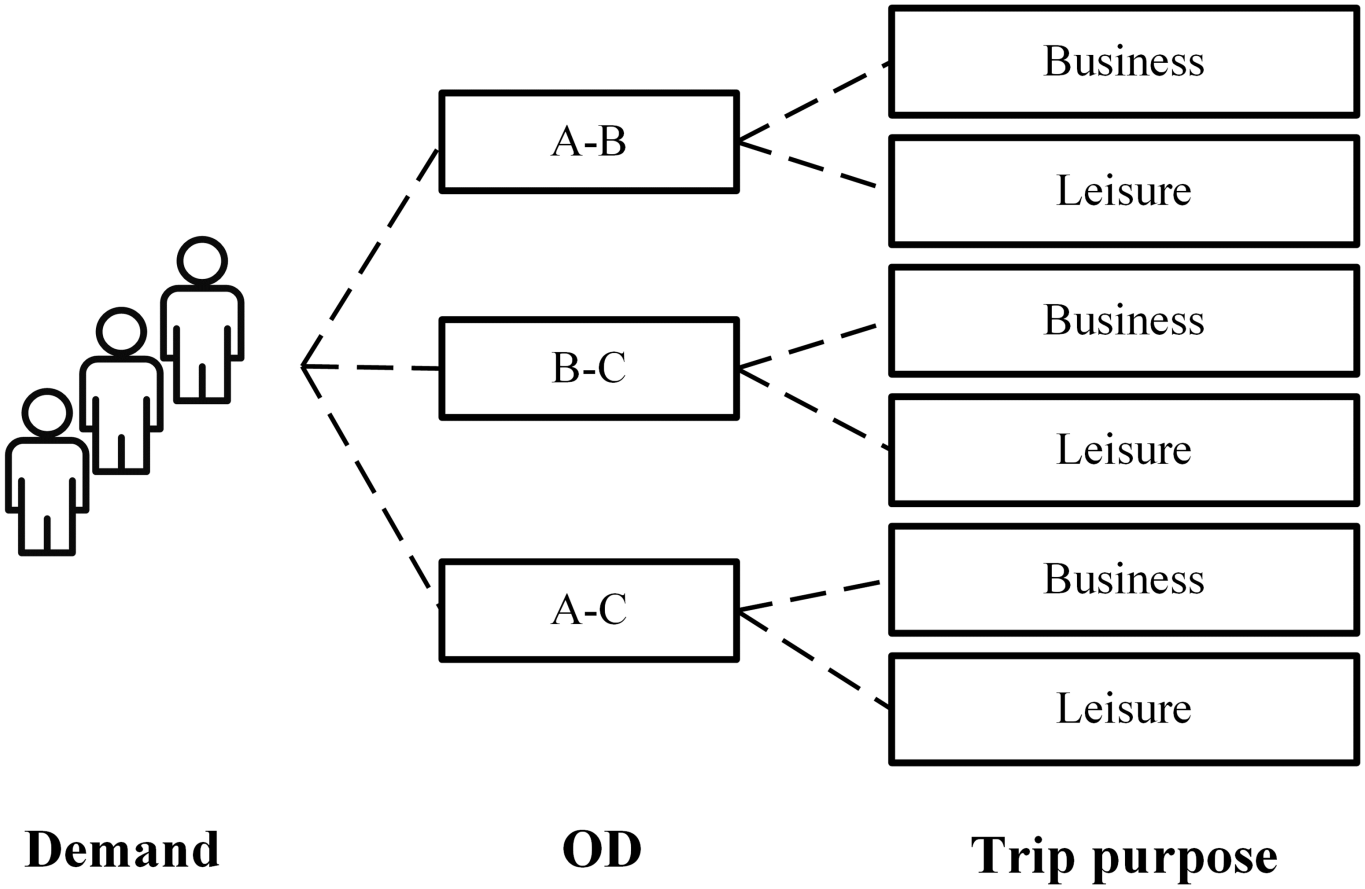
Market segmentation in the example network.

As mentioned in Section 1, to handle the heterogeneous passenger behavior, the entire market can be divided into several segments. A market segment is a group of customers with similar itinerary preferences. The first criterion of segmentation is origin-destination (OD). Because passengers of a specific OD have the same potential travel itineraries. The market can be further divided with trip purpose. For example, Fig 4 shows a market segmentation in the example network, in which each OD-level segment has a business subsegment and a leisure subsegment.

In this paper we use mutually-exclusive market segmentation [32], which means a passenger can just belong to one market segment. Thus each subsegment is independent. Let *l* denote a market segment, and the full market is denoted by *L*.

By segmentation, we can build a distinct passenger choice model for each market segment. The output of the passenger choice model is *P*_*l*,*k*_(*S*), which denotes the probability that a passenger from market segment *l* selects itinerary *k* from product set *S*. Here we use MNL model to model passenger choice for each market segment, which is formulated as (1). The MNL model can be substituted with any other passenger choice model, such as the finite-mixture logit model [14] and the ordered preference list (OPL) model [33].

$$P_{l,k}(S)=\frac{\beta_{k}^{l}}{\sum_{h\in K(l)\cap P(S)}\beta_{h}^{l}+\beta_{\emptyset }^{l}}$$ (1)

- $\beta_{k}^{l}$ is the preference weight for itinerary *k*, and $\beta_{\emptyset }^{l}$ denotes the no-purchase preference.
- *K*(*l*) ⊂ *K* denotes the consideration set of market segment *l*. If *k* ∉ *K*(*l*), $\beta_{k}^{l}=0$.
- P(S) represents the set of available itineraries supported by *S*.

**Fig 5:**
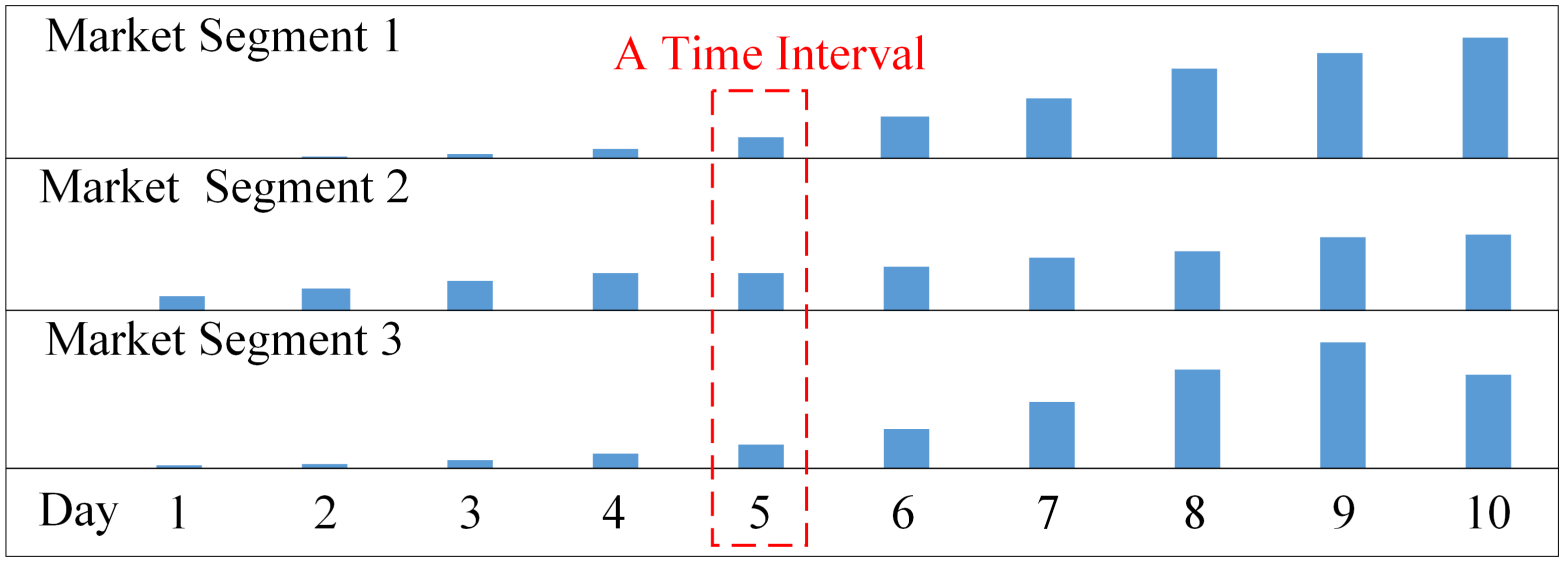
An example of daily passenger arrival forecasting.

The passenger arrivals are usually described by a Poisson process, whose rate is denoted *ρ*_*t*_. For a passenger arriving at time *t*, the probability that this passenger belongs to market segment *l* is denoted by λ_*t*,*l*_. The parameters (***ρ***, **λ**) can be derived from the demand forecasting results [11], whose typically form is the average passenger arrival amount of each market segment in a time interval [12, 34, 35]. Note that the length of each interval is not limited to one day; it can also be one hour or minute. Fig 5 shows an example of passenger arrival distribution over days. The vertical axis indicates estimated amount of passenger arrival in three segments; the horizontal axis indicates day of reservation horizon.

### 3.5 The bid-price-based seat inventory control problem

The aim of the dynamic bid price based seat inventory control problem is to determine the bid price *π*_*t*,*i*_ of each resource at discrete time period *t* = {1, …, *T*} that maximizes the expected revenue for the given network (*I*, *J*, *K*), initial state vector **c**, arrival parameter (***ρ***, **λ**) and passenger model for each segment.

The ticket-booking process is viewed as a finite-horizon and discrete-time process. The time index *t* starts at *t* = 1 and ends at *t* = *T*. Following Talluri and Ryzin [7], the bid price *π*_*t*,*i*_ can be calculated as the opportunity cost, which is formulated as *π*_*t*,*i*_ = *v*_*t*+1_ (**x**_*t*_) − *v*_*t*+1_ (**x**_*t*_ − **e**_*i*_). Here **e**_*i*_ is a unit vector of resource *i* (one for the dimension of resource *i* and zero for the other dimensions). *v*_*t*_(**x**_*t*_) represents the expected marginal revenue of the remaining **x**_*t*_ at time *t*. *v*_*t*_(**x**_*t*_) can be calculated using the DP formulation (2). (3) and (4) are the boundary conditions of the problem. The symbols are listed in Table 6.

$$\begin{array}{cc} \text{DP}: & \\ v_{t}(x_{t}) & =\underset{S\subset N(x_{t})}{\text{max}}\{\sum_{k\in K}P_{k}^{t}(S_{t})[\sum_{j\in k}f_{j}+v_{t+1}(x_{t}-A^{k})]+(1-\sum_{k\in K}P_{k}^{t}(S_{t}))v_{t+1}(x_{t})\}\\ & =\underset{S\subset N(x_{t})}{\text{max}}\{\sum_{k\in K}P_{k}^{t}(S_{t})[\sum_{j\in k}f_{j}-(v_{t+1}(x_{t})-v_{t+1}(x_{t}-A^{k}))]+v_{t+1}(x_{t})\}\\ & \;\forall t,x_{t}\in X \end{array}$$ (2)

$$\begin{array}{cc} & v_{t}(0)=0\;\;\;\forall t=1,\ldots ,T \end{array}$$ (3)

$$\begin{array}{cc} & v_{T+1}(x_{T+1})=0\;\;\;\forall x_{T+1}\in X \end{array}$$ (4)

**Table 6:** Symbol list.

Symbol	Description
*I*	The resource set, indexed by *i*
*J*	The product set, indexed by *j*
*K*	The itinerary set, indexed by *k*
*L*	The market segment set, indexed by *l*
*X*	The state space of state vector **x**
*N*	The action space of the product offer state, the power set of *J*
*T*	The time horizon, indexed by *t* = 1, …, *T*
*D*_*α*_	The set of time intervals before *t* = *α*, index by *d*
*S*_*t*_	The offer set at time *t*
*K*(*l*)	The consideration set of market segment *l*
*P*_*l*,*k*_(*S*)	The probability of a passenger from segment *l* choosing itinerary *k* with offer set *S*
$P_{k}^{t}(S)$	The probability of a passenger choosing itinerary *k* with offer set *S* at time *t*
P(S)	The set of available itineraries supported by *S*.
F(S)	The set of resources occupied by *S*.
*ρ*_*t*_	The probability of one arrival at time *t*
λ_*t*,*l*_	The probability of an arrival request belonging to segment *l* at time *t*
*f*_*j*_	The fare of product *j*
*v*_*t*_(**x**_*t*_)	The maximum expected revenue at time *t* with state vector **x**_*t*_
*π*_*t*,*i*_	the bid price of resource *i* at time *t*
*φ*(*d*)	The first time index in interval *d*.
*δ*(*t*)	The time interval which *t* belongs to.
**A**	The resource-itinerary matrix
**e**_*i*_	The unit vector of resource *i*
**x**_*t*_	The vector of the remaining units of all resources at time *t*
**c**	The initial vector of resources

- The set of offer products is denoted by *S*_*t*_ (called the offer set). *S*_*t*_ is feasible if there are sufficient remaining resources; that is, *S*_*t*_ ⊂ *N*(**x**_*t*_), where *N*(**x**_*t*_) = {*j*|*x*_*t*,*i*_ ≥ 1, ∀*i* ∈ *j*}.
- Let $P_{k}^{t}(S_{t})$ represent the probability that itinerary *k* is sold at time *t* with offer set *S*_*t*_. $P_{k}^{t}(S_{t})$ can be calculated by (5).
  $$\begin{array}{c} P_{k}^{t}(S_{t})=\sum_{l\in L}\rho_{t}\lambda_{t,l}P_{l,k}(S_{t}) \end{array}$$ (5)

**Fig 6:**
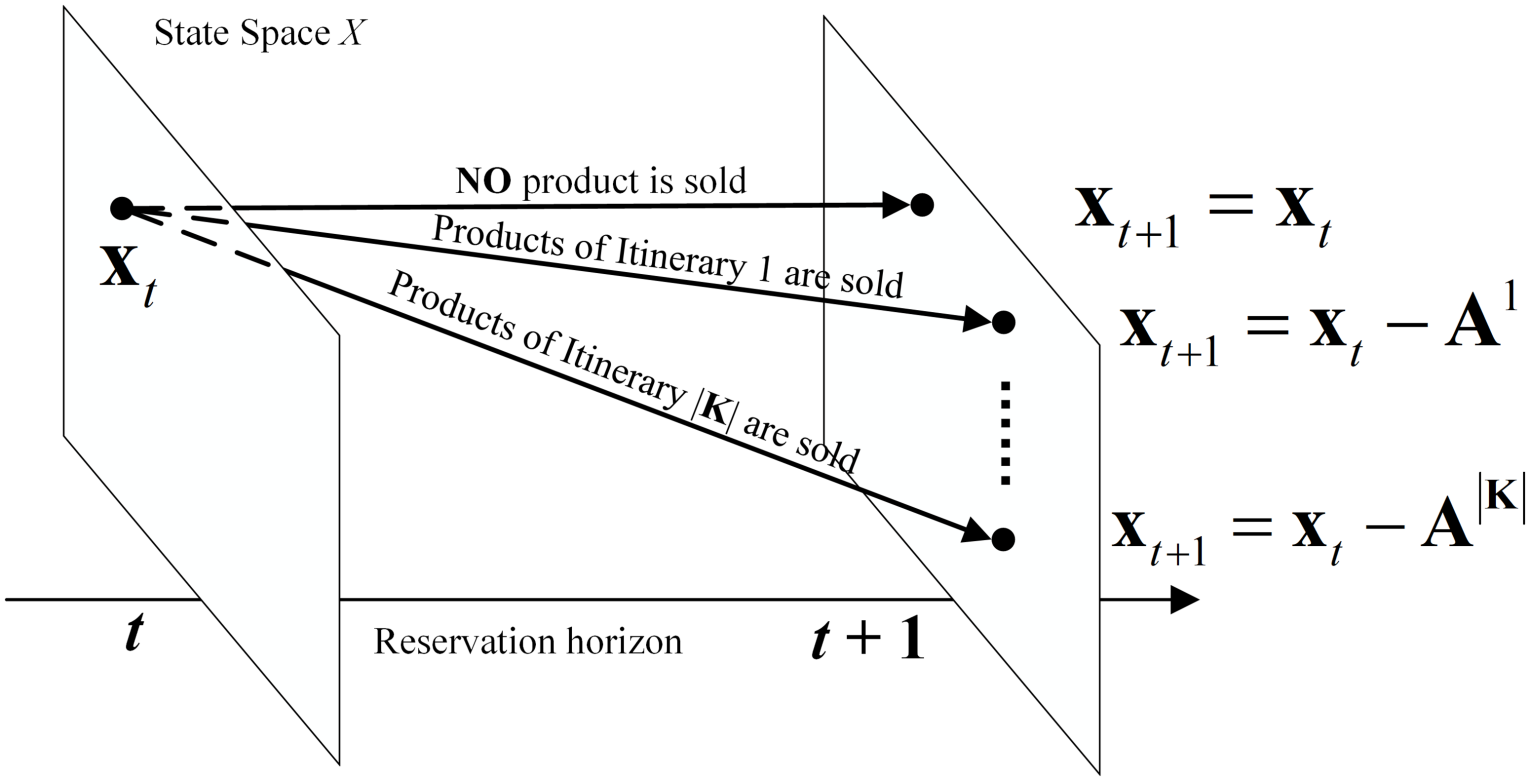
The structure of the DP model.

The Bellman Eq (2) provides a recursive way to estimate *v*_*t*_(**x**_*t*_). Fig 6 shows the structure of the DP model. At time *t*, for given **x**_*t*_, **x**_*t*+1_ has no more than |*K*| + 1 possibilities.

## 4 Solution methods

The DP model is intractable due to the large size of the state space *X* and action space *N*. In this section, we adapt linear-programming-based approximate dynamic programming (LP-based ADP) to solve the problem. This method was first introduced by Adelman [24] to compute bid prices. The basic idea of the method is to formulate an approximate affine function (6) of the value function *v*_*t*_(**x**_*t*_).

$$v_{t}(x_{t})\approx \theta_{t}+\sum_{i}\pi_{t,i}x_{i},\;\theta_{t}\geq 0,\pi_{t,i}\geq 0\;$$ (6)

The DP model can also be written in an equivalent LP form with an initial state vector **c**, in which the number of constraints is determined by the scale of *X* and *N* [36, 37].

$$\begin{array}{c} \text{LP}:\underset{\{v_{t}(\cdot )\}\;\forall t}{\text{min}}v_{1}(c)\\ \text{Subject}\;\text{to}\\ v_{t}(x_{t})\geq \sum_{k\in K}P_{k}^{t}(S_{t})[\sum_{j\in k}f_{j}-(v_{t+1}(x_{t})-v_{t+1}(x_{t}-A^{k}))]+v_{t+1}(x_{t})\;\\ \;\forall t,x_{t}\in X,S\subset N(x_{t}) \end{array}$$

After inserting (6) into LP, we get AF-LP. It has a good property that the bid prices are in the construction of its solution (*θ*, *π*), i.e., all the bid prices in the time horizon can be calculated by solving AF-LP.

$$\begin{array}{c} \text{AF}-\text{LP}:\;\underset{\theta ,\pi }{\text{min}}\;\theta_{1}+\sum_{i}\pi_{1,i}c_{i}\\ \text{Subject}\;\text{to}\\ \theta_{t}-\theta_{t+1}+\sum_{i}\pi_{t+1,i}Q_{t,i}(S_{t})+\sum_{i}(\pi_{t,i}-\pi_{t+1,i})x_{t,i}\geq R_{t}(S_{t})\;\;\forall t,x_{t}\in X,S_{t}\subset N(x_{t})\;\\ \theta ,\pi \geq 0 \end{array}$$ (7)

- For simplicity, let $R_{t}(S)=\sum_{k\in K}\sum_{j\in k}f_{j}P_{k}^{t}(S)$ and $Q_{t,i}(S)=\sum_{k\in K}a_{i,k}P_{k}^{t}(S)$.
- Specifically, we define *θ*_*T*+1_ = 0 and *π*_*T*+1,*i*_ = 0 ∀*i* according to the boundary conditions.

Compared to DP, the number of decision variables in AF-LP is decreased from *T* × |*N*| to *T* × (|*I*| + 1). However, the scale of the constraints (7) is still too large.

In Section 4.1, we introduce a reduced form of AF-LP, and in Section 4.2, we continue the reduction by using a dynamic disaggregated approach. Finally, in Section 4.3, we use heuristic methods to solve the subproblems of constraint generation.

### 4.1 Reduction of the state space

**Fig 7:**
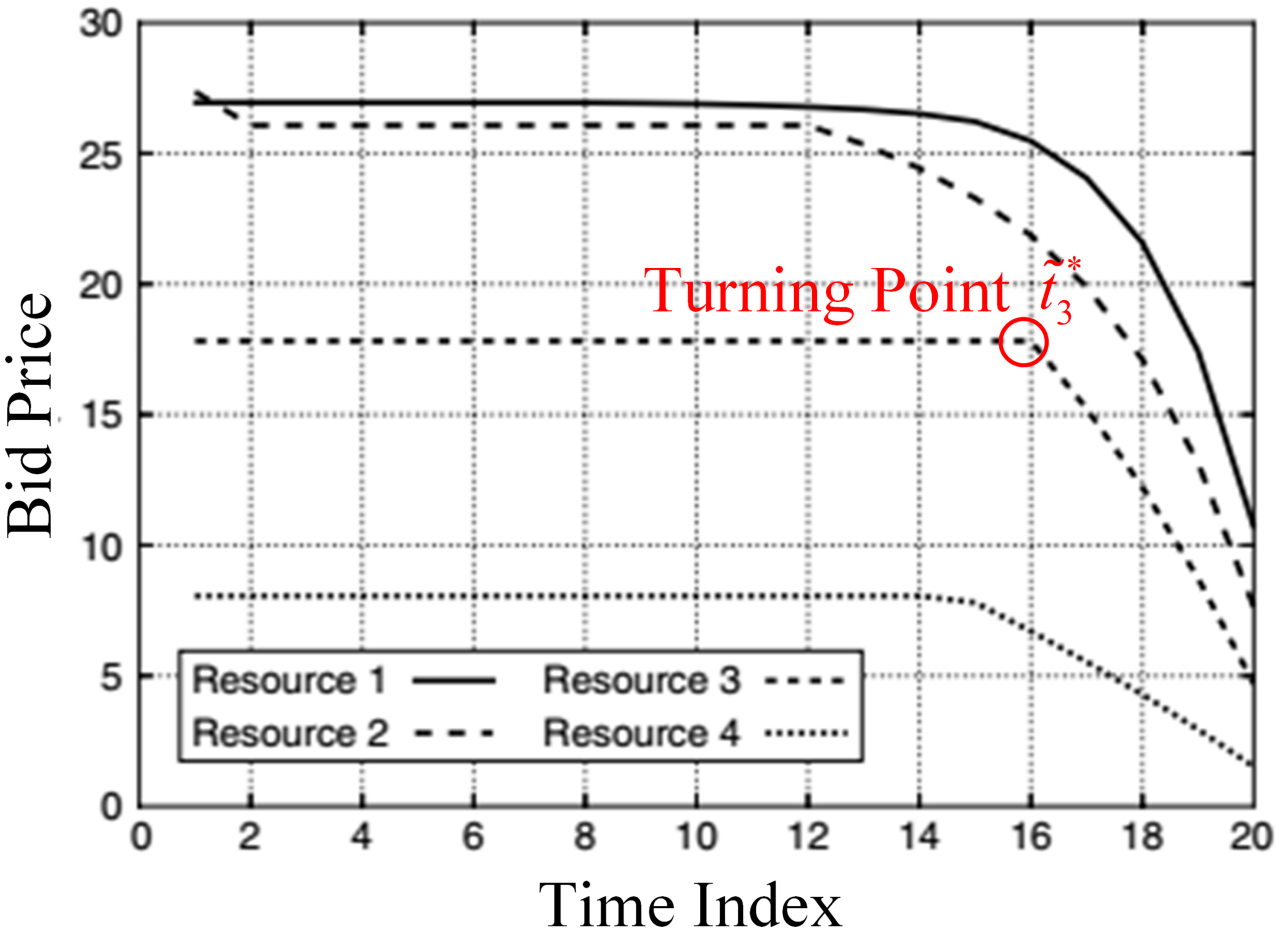
An example of dynamic bid price trajectories.

Zhang and Adelman [30] proposed Theorem 1 with complementary slackness, which implies that the optimal bid price *π*_*t*,*i*_ is monotonically decreasing with *t*, as shown in Fig 7.

**Theorem 1** *There exists an optimal solution* (θ*, π*) *of AF-LP and a set of indices* $\{{\tilde{t}}_{i}^{*}\}\;\forall i$ *such that* (8)–(11) *hold*.

$$\theta_{t}^{*}\geq \theta_{t+1}^{*}\;\forall t,$$ (8)

$$\pi_{t,i}^{*}=\pi_{t+1,i}^{*}\;\forall i,t=1,\ldots ,{\tilde{t}}_{i}^{*}-1$$ (9)

$$\pi_{t,i}^{*}\geq \pi_{t+1,i}^{*}\;\;\forall i,t={\tilde{t}}_{i}^{*},\ldots ,T$$ (10)

$$\theta^{*},\pi^{*}\geq 0$$ (11)

According to AF-LP, the cluster of constraints (7) for a given *t* and *S* cannot be tight simultaneously. If (θ*, π*) is an optimal solution of AF-LP, then from Theorem 1, we have $\pi_{t,i}^{*}-\pi_{t+1,i}^{*}\;\geq 0$. Note that ∀**x**_*t*_ ∈ *X*, *S*_*t*_ ⊂ *N* (**x**_*t*_) ⇔ ∀*S*_*t*_ ⊂ *J*, **x**_*t*_ ∈ *X* (*S*_*t*_), where *X*(*S*_*t*_) indicates the state that can support *S*_*t*_. For each time period *t*, (12) holds.

$$\begin{array}{c} \sum_{i}(\pi_{t,i}^{*}-\pi_{t+1,i}^{*})x_{t,i}\geq \sum_{i\in F(S)}(\pi_{t,i}^{*}-\pi_{t+1,i}^{*})\;\\ \forall S_{t}\subset J,x_{t}\in X(S_{t}) \end{array}$$ (12)

- F(S) represents the set of resources occupied by *S*.
  $$\begin{array}{c} F(S)=\{i|\sum_{j\in S}1_{\left\{i\in j\right\}}\geq 1,\;\forall i\in I\} \end{array}$$
- 1_{*i*∈*j*}_ is an indicator function, which equals 1 if *i* ∈ *j*; otherwise, it equals 0.

Therefore, the complexity of the state space of AF-LP can be reduced to RAF-LP. The equivalence of AF-LP and RAF-LP is proofed by Vossen and Zhang [27], where the reduced form is derived using Dantzig-Wolfe decomposition in the dual problem.

$$\begin{array}{c} \text{RAF}-\text{LP}:\;\underset{\theta ,\pi }{\text{min}}\;\theta_{1}+\sum_{i}\pi_{1,i}c_{i}\\ \text{Subject}\;\text{to}\\ \theta_{t}-\theta_{t+1}+\sum_{i\in F(S)}[\pi_{t,i}-\pi_{t+1,i}(1-Q_{t,i}(S))]\geq R_{t}(S)\;\forall t,S\subset J\\ \pi_{t,i}\geq \pi_{t+1,i}\;\forall t,i\\ \theta_{t}\geq \theta_{t+1}\;\forall t\\ \theta ,\pi \geq 0 \end{array}$$ (13)

The complexity of the state space is reduced in RAF-LP, which can be solved using the constraint generation method with subproblems (14) for every time index *t*. The remaining difficulty is the scale of horizon *T*, which leads to the large scale of the subproblems.

$$\begin{array}{c} \underset{S\subset N}{\text{max}}R_{t}(S)-\sum_{i\in F(S)}[\pi_{t,i}-\pi_{t+1,i}(1-Q_{t,i}(S))]-\theta_{t}+\theta_{t+1} \end{array}$$ (14)

### 4.2 Dynamic disaggregation approach

In Section 3.4, the arrival passenger stream within a time interval can be viewed as a homogeneous Poisson process, i.e., (*ρ*_*t*_, **λ**_*t*_) is constant. Therefore, *R*_*t*_(*S*) and *Q*_*t*,*i*_(*S*) are also constant. Theorem 1 shows that there is a turning point ${\tilde{t}}_{i}^{*}$ in the time sequence of the optimal bid price $\pi_{t,i}^{*}$. For $\alpha <\text{min}\;\{{\tilde{t}}_{i}^{*}\}\;\forall i$, (15) holds.

$$\begin{array}{c} \theta_{t}^{*}-\theta_{t+1}^{*}+\sum_{i\in F(S)}\pi_{t+1,i}^{*}Q_{t,i}(S)\geq R_{t}(S)\;\;\forall t\leq \alpha ,S\subset J \end{array}$$ (15)

Let *D*_*α*_ denote the set of intervals before *α* and indexed by *d*. Sum up the inequalities of (15) in time interval *d*. Let ${\bar{R}}_{d}(S)$ and ${\bar{Q}}_{d,i}(S)$ denote the identical *R*_*t*_(*S*) and *Q*_*t*,*i*_(*S*) in time interval *d*. Then, (16) holds.

$$\begin{array}{cc} & \frac{{\bar{\theta }}_{d}^{*}-{\bar{\theta }}_{d+1}^{*}}{\varphi (d+1)-\varphi (d)}+\sum_{i\in F(S_{t})}\pi_{\alpha +1,i}^{*}{\bar{Q}}_{d,i}(S_{t})\geq {\bar{R}}_{d}(S_{t})\;\forall d=1,\ldots ,D_{\alpha },S\subset J \end{array}$$ (16)

- *φ*(*d*) represents the first time index in interval *d*.
- Specifically, we define *φ*(*D*_*α*_ + 1) = *α* + 1 and ${\bar{\theta }}_{D_{\alpha }+1}^{*}=\theta_{\alpha +1}^{*}$.
- For simplicity, let ${\bar{\theta }}_{d}^{*}=\theta_{\varphi (d)}^{*}$.

By substituting (16) in for the constraints in RAF-LP, where *t* ≤ *α*, we construct RAF-LP-*α* as the aggregated form of RAF-LP.

$$\begin{array}{c} \text{RAF}-LP-\alpha :\;\underset{\theta ,\pi }{\text{min}}\;\theta_{1}+\sum_{i}\pi_{\alpha ,i}c_{i}\\ \text{Subject}\;\text{to}\\ \frac{{\bar{\theta }}_{d}-{\bar{\theta }}_{d+1}}{\varphi (d+1)-\varphi (d)}+\sum_{i\in F(S)}\pi_{\alpha +1,i}{\bar{Q}}_{d,i}(S)\geq {\bar{R}}_{d}(S)\;\forall d=1,\ldots ,D_{\alpha },S\subset J\\ \theta_{t}-\theta_{t+1}+\sum_{i\in F(S)}[\pi_{t,i}-\pi_{t+1,i}(1-Q_{t,i}(S))]\geq R_{t}(S)\;\forall t=\alpha \ldots T,S\subset J\\ \pi_{t,i}\geq \pi_{t+1,i}\;\;\forall t=\alpha ,\ldots ,T,i\\ {\bar{\theta }}_{d}\geq {\bar{\theta }}_{t+1}\;\;\forall d=1,\ldots ,D_{\alpha }\\ \theta_{t}\geq \theta_{t+1}\;\;\forall t=\alpha \ldots T\\ \theta ,\bar{\theta },\pi \geq 0 \end{array}$$

Let $\left(\theta ,\bar{\theta },\pi \right)$ be a solution of RAF-LP-*α*. We can construct a solution $\left(\tilde{\theta },\tilde{\pi }\right)$ of RAF-LP using (17) and (18)

$$\begin{array}{cc} {\tilde{\theta }}_{t} & =\{\begin{array}{cc} {\tilde{\theta }}_{\delta (t)}+\frac{{\tilde{\theta }}_{\delta (t)+1}-{\tilde{\theta }}_{\delta (t)}}{\varphi (d+1)-\varphi (d)}\cdot (t-\varphi (\delta (t))) & t=1,\ldots ,\alpha -1\\ \theta_{t} & t=\alpha \ldots T \end{array} \end{array}$$ (17)

$$\begin{array}{cc} {\tilde{\pi }}_{t,i} & =\{\begin{array}{cc} \pi_{\alpha +1,i} & t=1,\ldots ,\alpha -1\\ \pi_{t,i} & t=\alpha \ldots T \end{array} \end{array}$$ (18)

- *δ*(*t*) represents the time interval which *t* belongs to.

From this construction, it is obvious that RAF-LP-*α* is a relaxation of RAF-LP. Assume that $\alpha <\text{min}\;\{{\tilde{t}}_{i}^{*}\}\;\forall i$ and that $\left(\theta^{*},{\bar{\theta }}^{*},\pi^{*}\right)$ is an optimal solution of RAF-LP-*α*. The corresponding $\left(\tilde{\theta },\tilde{\pi }\right)$ gives an objective value for RAF-LP that is equivalent to the optimal objective of RAF-LP-*α*. Therefore, $\left(\tilde{\theta },\tilde{\pi }\right)$ is an optimal solution to RAF-LP if it is feasible. The feasibility of $\left(\tilde{\theta },\tilde{\pi }\right)$ is guaranteed because (15) and (16) are equivalent when *t* ≤ *α*.

The problem is then transformed into finding an $\alpha <\text{min}\;\{{\tilde{t}}_{i}^{*}\}\;\forall i$, which can be achieved by searching backwards from *α* = *T*. Vossen and Zhang [29] developed a DD approach based on this idea. We follow this idea and propose the following DD approach.

**Algorithm 1** Dynamic Disaggregated (DD) Approach

1: Initialization. Set *α* = *T* and initialize the search step *α*_*step*_ and tolerance *μ*.

2: Solution. Solve RAF-LP-*α* and record the objective value *V*_*α*_ and the corresponding $\left(\tilde{\theta },\tilde{\pi }\right)$.

3: Termination. If *α* = 1, solve RAF-LP and terminate. If *α* > 1 and *V*_*α*+1_ − *V*_*α*_ ≤ *μ*, terminate; $\left(\tilde{\theta },\tilde{\pi }\right)$ is an optimal solution. Otherwise, set *α* = max{1, *α* − *α*_*step*_} and return to Step 2.

The advantage of the DD approach is that it enables us to solve problems with a long time horizon and accelerates the solving process, except the worst case *α* = 1. The problem RAF-LP-1 is equivalent to RAF-LP.

### 4.3 Addressing the subproblems

RAF-LP-*α* can be solved by the constraint generation method; the subproblems are SUB-I and SUB-II. The two subproblems are combinatorial optimization problems with similar constructions. Note that the size of the action space *J* is 2^*n*^; therefore, it is impossible to enumerate all the actions for a large number of products. Two classes of methods are used in the current literature to determine the optimal *S* in the subproblems. The first is to use a heuristic approach to construct a solution [21, 33], and the second is to build a mathematical programming model that considers the structure of the choice model [30].

$$\begin{array}{cc} \text{SUB}-\text{I}: & \underset{S\subset N}{\text{max}}{\bar{R}}_{d}(S)-\sum_{i\in F(S)}\pi_{\alpha +1,i}{\bar{Q}}_{d,i}(S)-\frac{{\bar{\theta }}_{d}-{\bar{\theta }}_{d+1}}{\varphi (d+1)-\varphi (d)}\\ & \;\forall d=1,\ldots ,D\\ \text{SUB}-\text{II}: & \underset{S\subset N}{\text{max}}R_{t}(S)-\sum_{i\in F(S)}[\pi_{t,i}-\pi_{t+1,i}(1-Q_{t,i}(S))]-\theta_{t}+\theta_{t+1}\\ & \;\forall t=\alpha +1\ldots T \end{array}$$

In this paper, we follow Bront and Vulcano [21] and develop a simple greedy heuristic. Let *Value*(*S*) denote the objective function; this approach relates to both SUB-I and SUB-II.

**Algorithm 2** Simple Greedy Heuristic for Subproblems

1: Set *S* = ∅.

2: For each product *j* in *J*, if *Value*(*S* ∪ {*j*}) > *Value*(*S*), then *S* ≔ *S* ∪ {*j*}.

3: Repeat Step 2 until *S* is not modified or *S* = *J*.

## 5 Numerical experiments

In this section, we test the performance of our approach with two benchmarks and investigate the effectiveness of passenger transfer in different scenarios.

**Fig 8:**
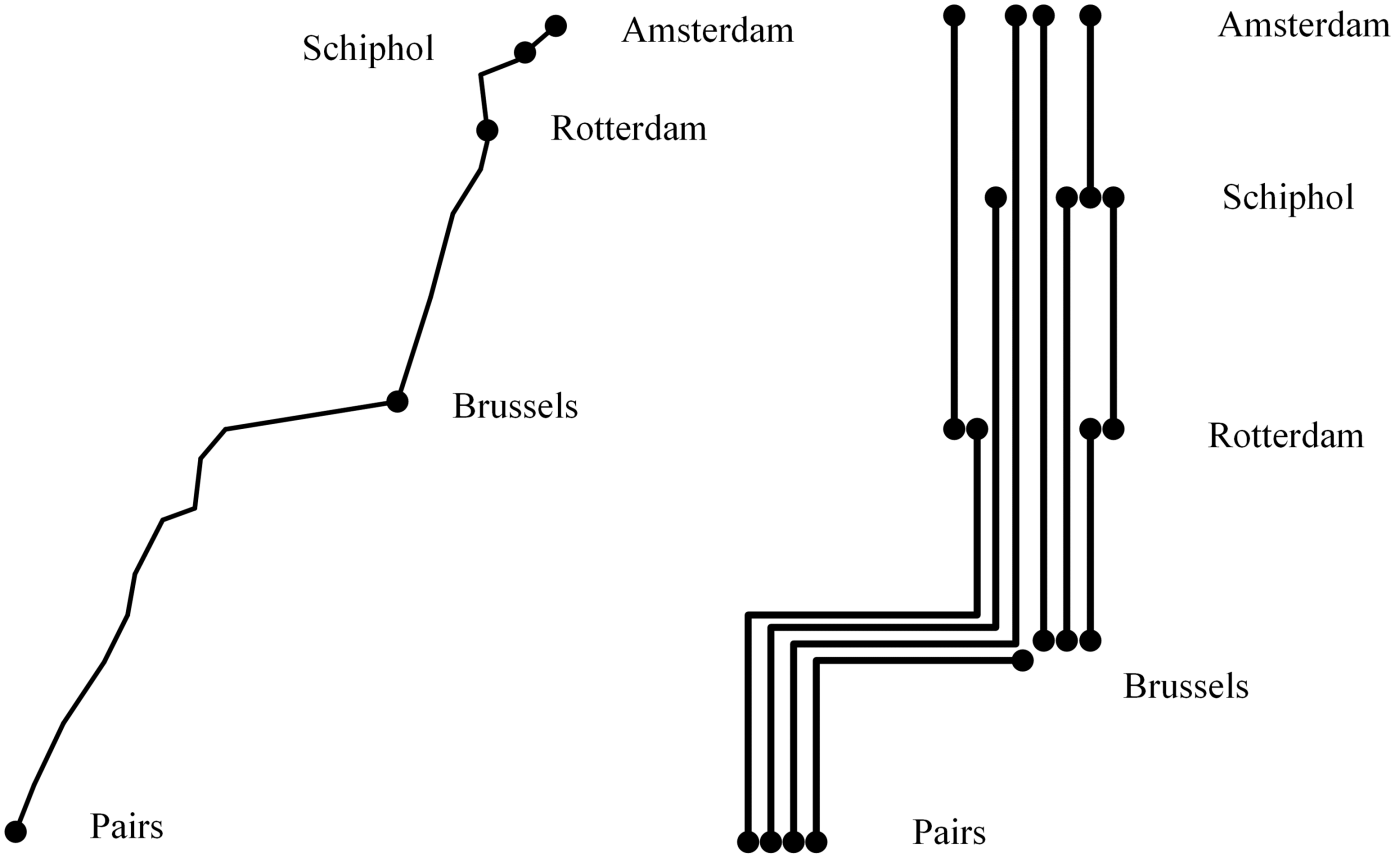
A map of Thalys high-speed railway.

The data for our numerical experiments is from a subnetwork of the Thalys high-speed railway system reported in Hosseinalifam et al. [22] (Fig 8). It includes five stations and ten trains. The products and market segments are given in Dataset S1 and S2 Datasets.

Our numerical experiments are conducted on a PC (2 cores of 3.00 G Hz, 2 GB RAM, Windows Server 2008 operating system). The algorithms are coded in C# and linked to the CPLEX 12.5 optimizer to solve the master problems.

### 5.1 The efficiency of our approach

In this section, we compare the performance of our approach with two alternative methods, CDLP and RAF-LP. Our interest is in the time cost and the expected revenue for different numbers of intervals. The CDLP method outputs a static bid price by solving an LP model under homogeneous arrival conditions. It runs fast relatively and can generate an upper bound of the expected revenue.

Here are the settings of this experiment:

- The values of *ρ*_*t*_ in 10 time intervals are in Table 7.
- The amount of time indexes in each interval is fixed to 100.
- λ_*t*,*l*_ is set to 0.05 for each market segment *l* ∈ *L*.
- The number of seats in a train is set to 5 (*c*_*i*_ = 5, ∀*i* ∈ *I*).

**Table 7:** *ρ*_*t*_ in all time intervals.

Interval	*ρ*_*t*_	Interval	*ρ*_*t*_
1	0.11	6	0.16
2	0.12	7	0.17
3	0.13	8	0.18
4	0.14	9	0.19
5	0.15	10	0.2

The results of four experiments are reported in Table 8. Note that in case 4, RAF-LP does not terminate within 48 hours. As can be seen from the results, our approach has the less processing time than RAF-LP and the same precision of the expected revenue as RAF-LP.

**Table 8:** Performance of our approach and two benchmarks.

#	Intervals	CDLP		RAF-LP		Our approach	
		Time(s)	Value	Time (s)	Value	Time (s)	Value
1	1	26	2,641	5,262	2,615	579	2,615
2	1-2	52	5,497	10,562	5,468	602	5,468
3	1-5	129	15,489	26,691	15,454	728	15,453
4	1-10	1,803	34,221	>48 h	-	4,315	33,829

### 5.2 The performance under different passenger transfer scenarios

**Fig 9:**
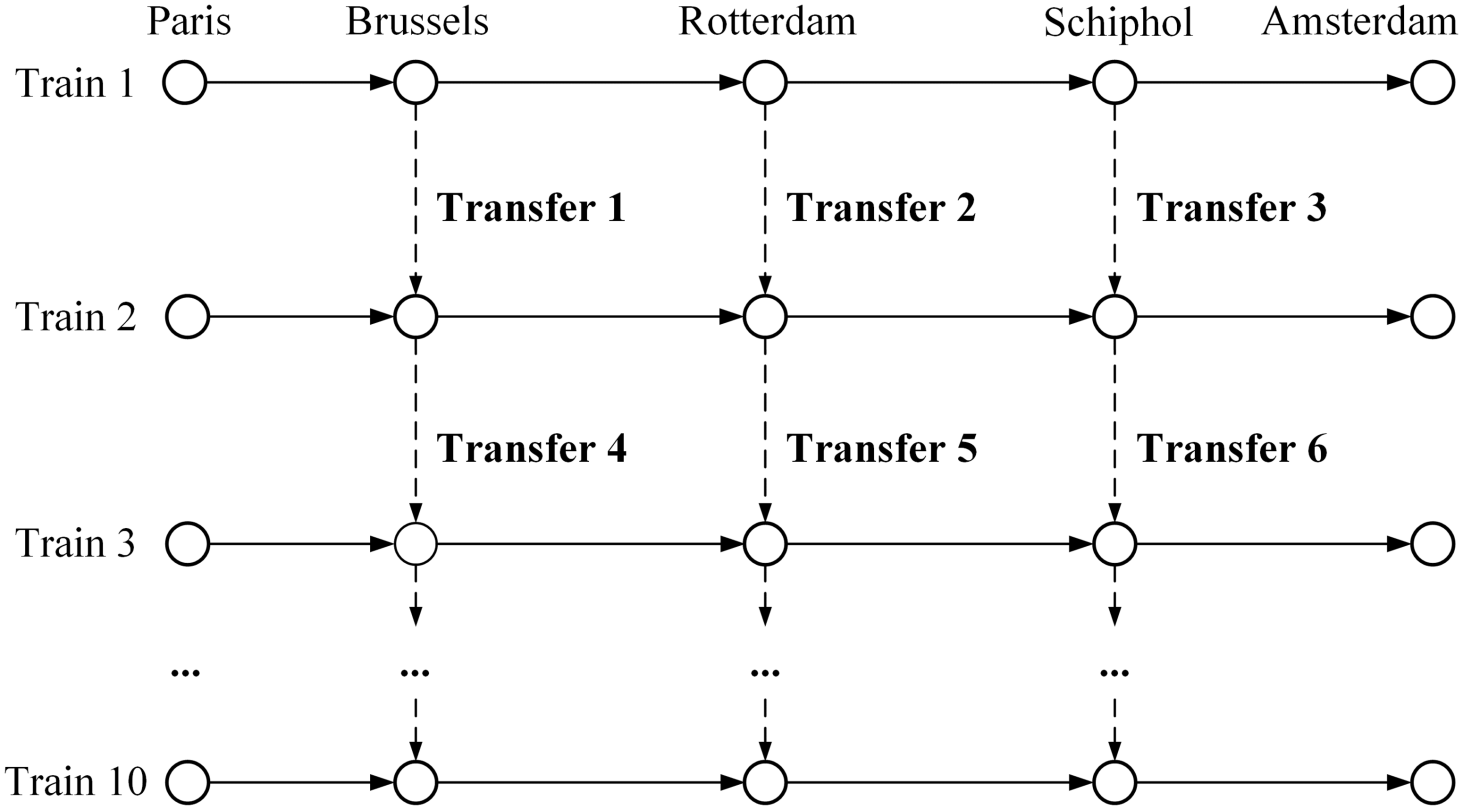
Available transfers.

The bid prices generated by our approach are tested in different transfer situations by simulation. To simplify the problem, we assume a passenger will transfer once at most. The transfer itineraries are shown in Fig 9. A passenger can transfer from one train to the next train via a transfer, for example, from train 1 to train 2 via transfer 1/2/3 or from train 2 to train 3 via transfer 4/5/6.

The transfer itineraries are numbered from 201 to 290 and added to the consideration set of the leisure market segments (i.e., segments 3, 5, 7, 11, 13, and 17). The preference of each itinerary is set to that of the direct product provided by the same train by which the initial product is supplied multiplied by a discount coefficient *β* ∈ [0, 1]. It indicates the willingness for transfer itineraries: passengers will be more likely to choose transfer when *β* increase. Table 9 shows an example of the additional itineraries with *β* = 0.5.

**Table 9:** Additional consideration set and preference vector with discount coefficient *β* = 0.5.

#	OD	Consideration Set	Preference Vector
3	PAR→RTA(L)	201…209	15,10,5,1.5,2.5,10,12.5,5,2
5	PAR→SCH(L)	210…236	12.5,10,2,2.5,2.5,2.5,3,3,5, 12.5,10,2,2.5,2.5,2.5,3,3,5
7	PAR→AMA(L)	219…254	10,1,2.5,2.5,3,3,3.5,3.5,3, 10,1,2.5,2.5,3,3,3.5,3.5,3, 10,1,2.5,2.5,3,3,3.5,3.5,3
11	BRU→SCH(L)	255…263	12.5,5,2.5,2.5,3,3,10,10,5
13	BRU→AMA(L)	264…281	12,2,2,1.5,1.5,2.5,3,3,5, 12,2,2,1.5,1.5,2.5,3,3,5
17	RTA→AMA(L)	282…290	20,5,2.5,2,1.5,2,2.5,2.5,3

The settings for this experiment are listed below.

- The values of *ρ*_*t*_ in 15 time intervals are in Table 10.
- The amount of time indexes in each interval is fixed to 1000.
- λ_*t*,*l*_ is set to 0.05 for each market segment *l* ∈ *L*.
- The number of seats in a train is set to 50 (*c*_*i*_ = 50, ∀*i* ∈ *I*).

**Table 10:** *ρ*_*t*_ in all time intervals.

Interval	*ρ*_*t*_	Interval	*ρ*_*t*_	Interval	*ρ*_*t*_
1	0.2	6	0.25	11	0.3
2	0.21	7	0.26	12	0.31
3	0.22	8	0.27	13	0.32
4	0.23	9	0.28	14	0.33
5	0.24	10	0.29	15	0.34

The experiments are conducted in 15 scenarios of 3 groups. In each scenario, we calculate a solution by using our approach and simulate the booking process. As a reference, simulations with free of control (FOC), which will offer any product as long as there are sufficient resources (i.e., all the bid prices are set to zero), are processed. The simulation of each scenario is repeated for 20,000 times.

The results are reported in Table 11. The description of its columns is listed below.

- Column UB represents the expected revenue *v*_1_(**c**), which is calculated by our approach.
- Columns BPC and FOC are the average revenue of simulations under bid price control and free of control.
- Column *γ* represents the load factor [30], which is an index of supply and demand. The load factor is calculated by (19), where $S^{*}\in \underset{S\subset N}{\text{arg}\;\text{max}}\underset{j\in S}{\sum }P_{j}(S)f_{j}$.
  $$\gamma =\frac{\sum_{t}^{\tau }\rho_{t}\sum_{k\in S^{*}}\sum_{i}a_{ik}P_{k}(S^{*})}{\sum_{i}c_{i}}$$ (19)

**Table 11:** Results of the simulation.

Group	Intervals	*γ*	Scenario	*β*	UB	BPC	FOC
1	1-5	0.9	1	0%	268,330	245,640	241,468
1	1-5	0.9	2	25%	268,932	246,642	241,650
1	1-5	0.9	3	50%	269,501	243,222	241,502
1	1-5	0.9	4	75%	269,974	246,130	241,221
1	1-5	0.9	5	100%	270,499	247,928	241,269
2	1-10	2.1	6	0%	364,659	322,542	289,857
2	1-10	2.1	7	25%	366,972	331,810	288,703
2	1-10	2.1	8	50%	368,453	302,965	287,883
2	1-10	2.1	9	75%	370,544	323,845	287,283
2	1-10	2.1	10	100%	363,596	325,207	287,328
3	1-15	3.5	11	0%	416,463	327,537	308,457
3	1-15	3.5	12	25%	419,284	336,568	306,760
3	1-15	3.5	13	50%	414,605	320,615	305,702
3	1-15	3.5	14	75%	410,818	354,766	304,846
3	1-15	3.5	15	100%	415,310	369,910	304,871

An intuitive observation is that the average revenue of bid price control is higher than free of control. The increase values of each scenarios are listed in Table 12. It indicates the increase is more evident in high-load-factor situations, as in Group 2 (*γ* = 2.1) and Group 3 (*γ* = 3.5).

**Table 12:** Increase of average revenue.

Group 1		Group 2		Group 3	
Scenario	Increase	Scenario	Increase	Scenario	Increase
1	4,172	6	32,685	11	19,080
2	4,992	7	43,107	12	29,808
3	1,720	8	15,082	13	14,913
4	4,909	9	36,562	14	49,920
5	6,659	10	37,879	15	65,039

**Fig 10:**
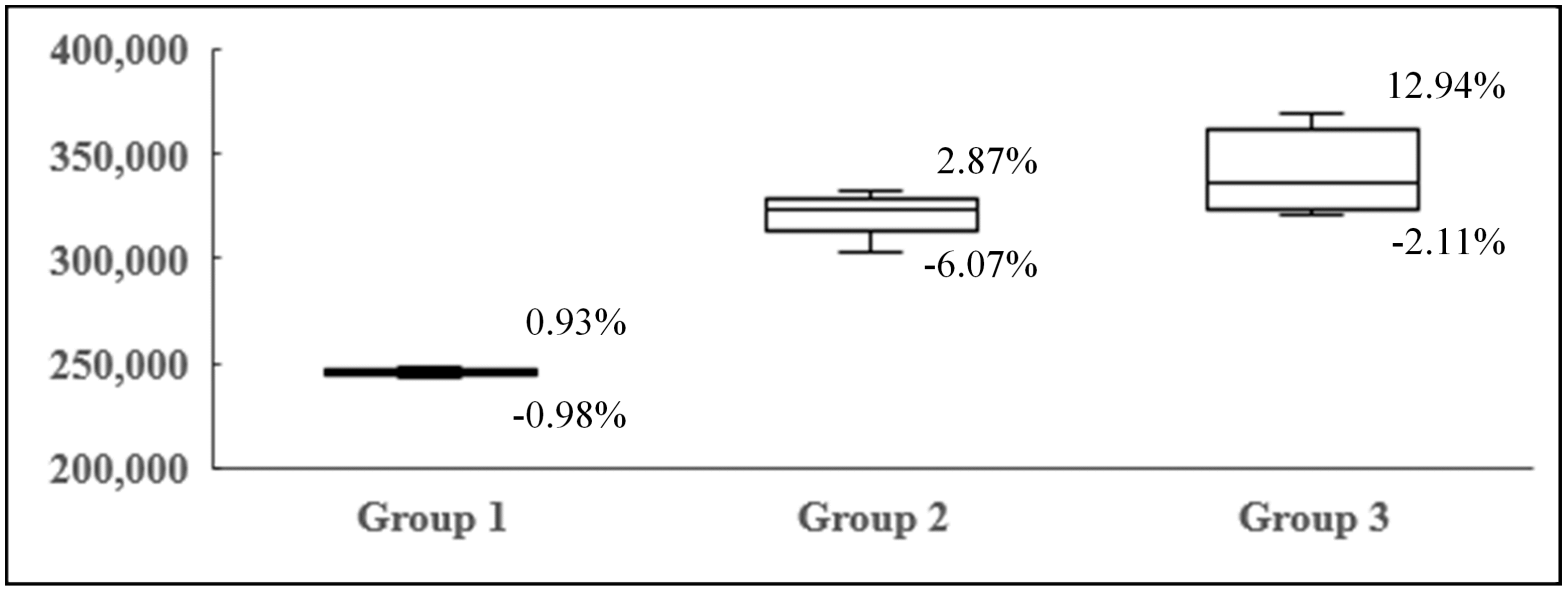
The box-plot of revenue in each group.

To verify the effect of passenger transfer, the fluctuation in the average revenue as a result of the change in *β* is shown in Fig 10. It can be clearly seen the strength of the fluctuation rises with the load factor. It also indicates passenger transfer should be seriously treated when the demand is relatively strong.

**Fig 11:**
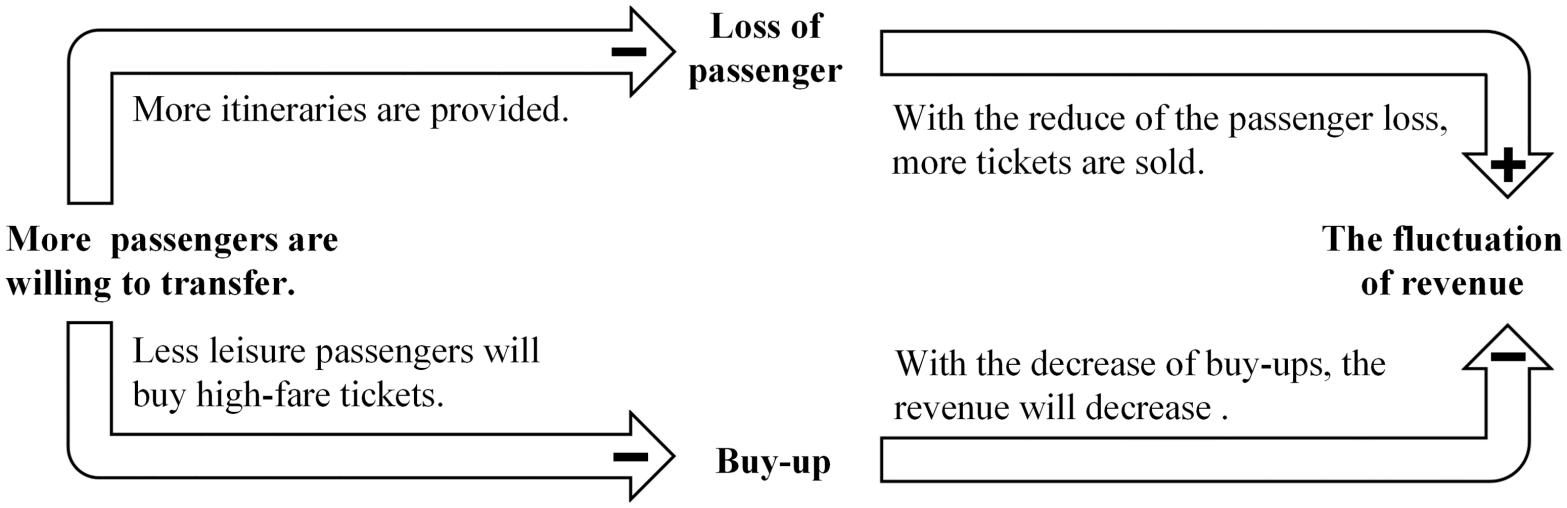
The two-sided effect of transfers on revenue.

The other interesting observation is that the average revenue is not monotonic increasing with *β*. The major reasons of the fluctuation are two-fold. Firstly, there are two contrary causal chains between transfer and revenue, as shown in Fig 11. With increasing preference for transfers, more products are sold as the only available itineraries between some ODs when the direct itineraries are sold out. On the other hand, transfers decrease the probability of a passenger in the low-market segment buying a high-fare product (buy-up). Besides, the heuristic methods we use to solve the subproblems may lead to the uncertainty in the quality of the solutions of the subproblems, and further have some influence on the optimality the bid prices.

Four indicators are calculated to investigate the impact of transfers from multiple perspectives in Table 13.

- **The percentages of passenger loss**
  The percentage of the passenger who give up traveling.
- **Transfer passengers**
  The percentage of the passengers who choose a transfer itinerary.
- **Transfer revenue**
  The percentage of the revenue created by transfer passengers.
- **High-fare product revenue**
  The percentage of the revenue by selling high-fare tickets in Appendix.

**Table 13:** Four indicators from ticket selling results.

#	Passenger Loss	Transfer Passenger	Transfer Revenue	High-Fare Product Revenue
1	20.33%	-	-	77.20%
2	20.12%	3.88%	4.42%	75.98%
3	20.06%	8.88%	9.87%	71.19%
4	20.09%	8.31%	9.51%	73.47%
5	20.05%	9.41%	10.88%	72.70%
6	54.23%	-	-	94.46%
7	52.10%	0.24%	0.09%	94.50%
8	54.10%	0.24%	0.10%	94.98%
9	53.13%	0.01%	0.02%	95.46%
10	52.07%	1.15%	1.36%	93.00%
11	70.06%	-	-	96.92%
12	69.40%	0.01%	0.01%	97.61%
13	71.61%	0.02%	0.02%	97.68%
14	69.99%	0.01%	0.01%	99.50%
15	69.43%	0.01%	0.02%	97.83%

From Table 13, the percentage of transfer passengers goes down with high load factor. In contrast, the percentage of high-fare products goes up. It is an obvious ascend number of transfer passenger with *β* in Group 1. It reflects the pattern of passenger flows are changed when the load factor is low.

### 5.3 Notes for experiments

Note that in the experiments, we reduced the scales of the supplies (amount of the trains and the seats) and the demand at the same proportion of real-world situations. To verify the applicability and efficiency of our method, we test a case in which the number of seats in train is set to 1000 and the demand is increased until the load factor is close to 2. The running time is nearly 5 hours. Although it is almost 10 times longer than the solving time of the small cases, it is still acceptable for a railway operator. Because usually the bid prices are determined before the reservation horizon starts so that they have enough time to calculate.

## 6 Conclusion

In this paper, we study the application of dynamic bid price control in railway seat inventory control problem. We build a DP model to compute dynamic bid prices consideration of multi-dimensional demand.

The randomness of passenger arrival (both amount and time) is modeled as a non-homogeneous passenger arrival process. We extend the dynamic disaggregated approach proposed by Vossen and Zhang [29] to compute bid prices in the non-homogeneous situation. The numerical experiments reports that our approach can significantly shorten the solving time without loss of accuracy.

The possible impact of transfer on revenue is verified by simulations. And the revenue fluctuation grows with the increase of load factor. In our cases, the fluctuation range from -2.11% to +12.94%. Besides, we find that transfer will affect the pattern of passenger flows when the load factor is relatively low.

## Supporting information

S1 Dataset Market segments data for the numerical experiments. (XLSX)

S2 Dataset Products data for the numerical experiments. (XLSX)
